# Eruption of Bioengineered Teeth: A New Approach Based on a Polycaprolactone Biomembrane

**DOI:** 10.3390/nano11051315

**Published:** 2021-05-17

**Authors:** Céline Stutz, François Clauss, Olivier Huck, Georg Schulz, Nadia Benkirane-Jessel, Fabien Bornert, Sabine Kuchler-Bopp, Marion Strub

**Affiliations:** 1 INSERM (French National Institute of Health and Medical Research), UMR 1260, CRBS Regenerative NanoMedicine (RNM), FMTS, 1 rue Eugène Boeckel, 67084 Strasbourg, France; celine.stutz@etu.unistra.fr (C.S.); francois.clauss@chru-strasbourg.fr (F.C.); o.huck@unistra.fr (O.H.); nadia.jessel@inserm.fr (N.B.-J.); fabien.bornert@unistra.fr (F.B.); kuchler@unistra.fr (S.K.-B.); 2 Faculty of Dentistry, University of Strasbourg (UDS), 8 rue Ste Elisabeth, 67000 Strasbourg, France; 3 Department of Pediatric Dentistry, University Hospitals of Strasbourg (HUS), 1 Place de l’Hôpital, 67000 Strasbourg, France; 4 Department of Periodontology, University Hospitals of Strasbourg (HUS), 1 Place de l’Hôpital, 67000 Strasbourg, France; 5 Core Facility Micro- and Nanotomography, Biomaterials Science Center (BMC), Department of Biomedical Engineering, University of Basel, Gewerbestrasse 14, 4123 Allschwil, Switzerland; georg.schulz@unibas.ch; 6 Department of Oral Medicine and Oral Surgery, University Hospitals of Strasbourg (HUS), 1 Place de l’Hôpital, 67000 Strasbourg, France

**Keywords:** biomembrane, electrospinning, tooth bioengineering, tooth eruption

## Abstract

Obtaining a functional tooth is the ultimate goal of tooth engineering. However, the implantation of bioengineered teeth in the jawbone of adult animals never allows for spontaneous eruption due mainly to ankylosis within the bone crypt. The objective of this study was to develop an innovative approach allowing eruption of implanted bioengineered teeth through the isolation of the germ from the bone crypt using a polycaprolactone membrane (PCL). The germs of the first lower molars were harvested on the 14th day of embryonic development, cultured in vitro, and then implanted in the recipient site drilled in the maxillary bone of adult mice. To prevent the ankylosis of the dental germ, a PCL membrane synthesized by electrospinning was placed between the germ and the bone. After 10 weeks of follow-up, microtomography, and histology of the implantation site were performed. In control mice where germs were directly placed in contact with the bone, a spontaneous eruption of bioengineered teeth was only observed in 3.3% of the cases versus 19.2% in the test group where PCL biomembrane was used as a barrier (*p* < 0.1). This preliminary study is the first to describe an innovative method allowing the eruption of bioengineered tooth implanted directly in the jawbone of mice. This new approach is a hope for the field of tooth regeneration, especially in children with oligodontia in whom titanium implants are not an optimal solution.

## 1. Introduction

The replacement of teeth lost due to congenital, infectious, or traumatic reasons is required to restore masticatory function and the aesthetics of the smile. Today, dental implants are considered one of the major treatment options [1]. However, as they are made of inert material, such as titanium or zirconia, there is only the development of a limited vascular nor nervous system decreasing associated sensitivity and increasing the risk of biological complications [2]. Nevertheless, implant placement is contraindicated for patients having been treated with radiotherapy in the head or neck region with doses higher than 50 Gy or with bisphosphonates [3]. Implant placement is also contraindicated in young patients, for instance, those with anodontia, where early implant placement does not allow the implant to follow bone growth, thus postponing extensive implant rehabilitation into adulthood [4,5]. During this period of time, the standard treatment consists of an implant-supported removable denture. In this situation, the bone support is reduced due to the absence of teeth, while the placement of bioengineered tooth germs would be accompanied by the formation of alveolar bone and *periodontium*. Tooth bioengineering is relevant if the obtained tooth presents the appropriate morphology, vascularized and innervated pulp tissue, and if the tooth is functional after its eruption in the oral cavity [6]. Several techniques have been suggested for tooth bioengineering with or without the use of a scaffold to guide the formation of the different tissues and to control morphogenesis [7]. In scaffold-free approaches, the different tissues spontaneously organize themselves to form dentin, enamel, and pulp tissue due to the differentiation properties of the stem cells and the influence of various growth factors [7]. The only team that regenerated a functional tooth and positioned it directly on the dental arch used a preliminary implantation step in the kidney [8]. In our previous work on whole tooth regeneration from embryonic dental germs or epithelial-mesenchymal reassociations, the tooth eruption rarely occurred [9]. The implanted germ was growing in the jawbone. Vascular tissue was detected in the pulp cavity, and additive techniques were able to stimulate the growth of peripheral nerve fibers associated with the blood vessels [9,10,11]. However, with these approaches, the bioengineered tooth remained within the bone crypt, under the oral mucosa, and therefore was not functional.

Theories explaining the mechanisms of tooth eruption in physiological conditions have constantly evolved, with specific emphasis made on each of the dental germ and *periodontium* components (bone, periodontal ligament, root...). Currently, authors agree that tooth eruption is controlled by the interactions between dental and surrounding tissues orchestrated by the spatial and temporal expression of growth factors [12]. In mice, the development kinetics are relatively synchronous, between the mandibular first molars and the maxillary first molars. At birth, molars have not yet erupted in the oral cavity. From the 10^th^ postnatal day (PN10), movements of the tooth germ in the occlusal direction place it under the oral epithelium [13]. A dental eruption path is created by the resorption of the alveolar bone in response to the activation of osteoclasts. Apposition of bone tissue takes place in the apical region, especially around furcation. Root formation begins while the germ is still in its bony crypt. The soft tissue becomes thinner, and the oral epithelium fuses with the enamel organ. Apoptosis of the soft tissues next to the cusps allows the tooth to pass through the mucosa at PN16 [13]. At PN20, the crown is fully visible in the oral cavity and in contact with the antagonist molar. The absence of eruption is the main limitation of tooth bioengineering. This lack of eruption is mainly associated with ankylosis of root due to the lack of molecular signaling. However, in most of the studies, the germs were implanted in adult mice at sites where no teeth are supposed to erupt. Therefore, the bone architecture is different from alveolar bone as well as molecular signaling.

Polycaprolactone (PCL) is a synthetic polymer often used in tissue engineering due to its biocompatibility, resorbability, and its mechanical properties [14]. Indeed, a PCL-based biomembrane obtained by electrospinning allows mimicking the extracellular matrix to promote cell adhesion [15,16]. During PCL biomembrane synthesis, various parameters (solvent, polymer solution, processing parameters, humidity percentage, and temperature) are used to control the structure and alignment of the electrospun nanofibers, thus modifying its biophysical properties [17]. The interactions between the cells and the membrane are facilitated by the large surface exposed due to the nanoporosity. Indeed, electrospun nanofibers can also enhance angiogenesis depending on pore size [18]. PCL-based electrospun nanofibers are the most used nanofibrous mats as angiogenic biomaterials. This scaffold allowed for the infiltration of pro-angiogenic growth factors, including VEGF-D. PCL has the advantage of being able to be associated with other components, such as BSA, which improves cell adhesion to nanofibers [19], or VEGF, to improve angiogenesis. It can be used as a mechanical barrier, but it can also be functionalized with active molecules, such as growth factors, to stimulate the eruption or innervation of the tooth [11,20].

The aim of this work was to develop an innovative approach allowing the eruption of implanted bioengineered tooth. We selected not to act on the signaling pathways but to compensate for these interactions by placing a mechanical barrier at the interface between the germ and the bone, preventing or slowing down ankylosis processes. The criteria were that the intervention should be technically simple, fast, and no more invasive than the implantation of the germ alone, with a biocompatible, thin, and deformable material that can optionally be functionalized with molecules of interest in a second step. Herein, we hypothesize that the placement of a biocompatible PCL membrane to isolate the germ may be an interesting strategy to promote tooth eruption.

## 2. Materials and Methods

### 2.1. Materials

Polycaprolactone (MW 124 kDa) clinical grade was purchased from Corbion (Gorinchem, The Netherlands).

### 2.2. Preparation of Electrospun Nanofibers

PCL was dissolved in a mixture of dichloromethane/dimethylformamide (DCM/DMF 50/50 *v/v*) at 15% *wt/v* and stirred overnight before use. A standard electrospinning set-up (EC-DIG apparatus, IME Technologies, Eindhoven, Netherlands) was used to synthesize the PCL scaffolds. The PCL solution was poured into a 5 mL syringe and ejected through a needle with a diameter of 0.5 mm at a flow rate of 1.2 mL·h^−1^ with a programmable pump (Harvard apparatus). A high-voltage power supply (SPELLMAN, SL30P10) was used to set 15 kV at the needle. The humidity percentage (RH%) and the temperature in the chamber were maintained and controlled respectively at 45 ± 5% and 19 ± 2 °C. Aluminum foils (20 × 20 cm²), connected to the ground collector at a distance of 17 cm from the needle, were used to collect the electrospun PCL scaffold. PCL scaffolds were dried in a vacuum oven overnight to remove traces of solvent.

### 2.3. Morphological Characterization of Electrospun Nanofibers

**Figure 1:**
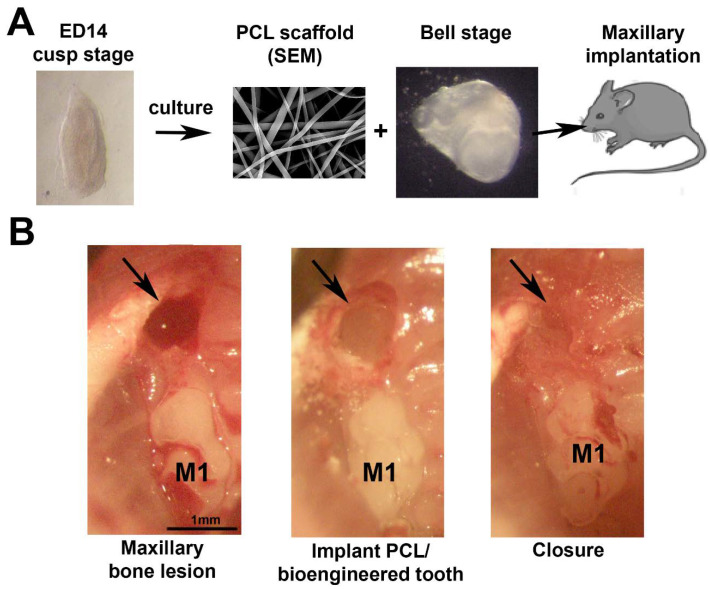
Study protocol. (**A**) Bioengineered tooth (bell stage) preparation and implantation in the maxillary bone. (**B**) Stages of the microsurgery: maxillary bone lesion obtained with a dental bur (500 μm), implantation of the PCL membrane with the bioengineered tooth, and closure of the gingiva with biological glue. ED14 = Embryonic Day 14, M1 = first molar, SEM = scanning electronic microscopy.

SEM allowed to characterize the morphological structure of the nanofibers (Figure 1A). Biomembranes were fixed and dehydrated in ethanol baths of increasing concentration (25%, 50%, 75%, 90%, and 100%) for 15 min each. They were placed on a specimen holder and fixed with carbon-conductive adhesive tape. Hexamethyldisilazane (HDMS, ThermoFisher Scientific, Illkirch, France) was deposited on the samples.

### 2.4. Tooth Bioengineering

The germs of the first mandibular molar (M1) were collected on the 14th day of embryonic development (ED14). They were cultured for five days on a semi-solid medium composed of 15 mL of DMEM-F12, 0.2 mL of vitamin C (Merck, Lyon, France), 0.2 mL of glutamine (Invitrogen, Villebon sur Yvette, France), 0.2 mL of a mixture of penicillin and streptomycin (Invitrogen, Villebon sur Yvette, France), 4 mL of fetal bovine serum (FBS) and agar diluted in sterile water. The medium was changed every two days until the bell stage of development (Figure 1A).

### 2.5. In Vivo Micro-Surgical Protocol

The experimental protocol fulfilled the authorization of the “Ministère de l’Enseignement Supérieur et de la Recherche” under the agreement numbers 01716.02 and APAFIS#269262020080410295545. The Ethics Committee of Strasbourg named “Comité Régional d’Ethique en Matière d’Expérimentation Animale de Strasbourg (CREMEAS)” specifically approved this study. All procedures were performed under anesthesia by intraperitoneal injection of a mixture of ketamine and xylazine (100 mg/kg of ketamine and 10 mg/kg of xylazine). Following a gingival incision in the diastemal area, two maxillary bone lesions of 500 µm were made with a dental bur (Figure 1B). In one of them, a dental germ harvested at ED14 and cultured in vitro was implanted, and in the second one, a PCL membrane and one dental germ were implanted. Then, the gingiva was closed with biological glue (Histoacryl^®^, B. Braun, Rubi, Spain). Mice were sacrificed, and samples were collected after 3 to 10 weeks of implantation. The jaws were preserved overnight in 4% PFA at 4 °C.

### 2.6. Microtomography

The samples recovered after different implantation times (3–10 weeks) were fixed overnight in 4% PFA in PBS at 4 °C and then immobilized in agar in a 15 mL tube. X-ray microCT acquisitions were performed using the Nanotom^®^ m system (GE Sensing & Inspection Technologies GmbH, Wunstorf, Germany), and images were generated with a reconstructed isotropic voxel size of 8 μm. 3D isosurface images were performed with Microview (Parallax Innovations Inc., Ilderton, ON, Canada).

### 2.7. Histology

Upper jaws were harvested and fixed for 24 h in 4% PFA in PBS, decalcified in Decalcifier II (Leica Microsystems, Nanterre, France) at 37 °C for 2 h under agitation and embedded in paraffin. Serial sections (10 μm) were stained with trichrome dyes of Gomori and Mallory and then observed with a Leica DM4000B microscope.

### 2.8. Statistical Analysis

A total of 28 animals were used for this pilot study, and for each mouse, an implantation was performed at both sides of the maxillary jaw. Fisher t-test was used for statistical analysis. Significance was considered for a *p*-value < 0.05.

## 3. Results

### 3.1. Characterization of the Biomembrane

The nanofibrous structure of the membrane was characterized by scanning electron microscopy (SEM). The SEM analysis of the membrane showed that the fibers were uniform in size and interconnected in order to mimic the natural extracellular matrix (Figure 1A). The mean diameter of fibers was 374 +/− 89 nm.

### 3.2. Tooth Development

**Figure 2:**
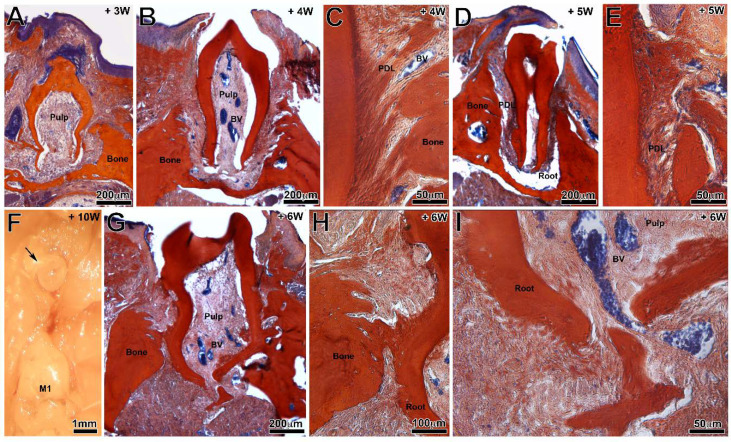
Histological sections of bioengineered teeth implanted with (**B**,**C**,**E**,**F**,**G**–**I**) or without a PCL membrane (**A**,**D**) at 3 (**A**), 4 (**B**,**C**), 5 (**D**,**E**) and 6 (**G**–**I**) postoperative weeks (Mallory staining). (**F**) Binocular observation of bioengineered tooth eruption after 10 weeks of implantation. BV = blood vessel, PDL = periodontal ligament.

Among the implanted germs, 60.7% continued their development after implantation (3 to 10 weeks). However, no significant difference was observed between groups in terms of tooth formation. Indeed, 69.2% and 53.3% of the germs formed teeth in the test and control group respectively (*p* > 0.05). The newly formed molars were of appropriate morphology, with a crown with several cusps (Figure 2A,B,D,G) and an early root formation (Figure 2G–I). Periodontal ligament was visible between root and maxillary bone (Figure 2C,E). All teeth were smaller than the maxillary first molar adjacent to the implantation site, with a reduction in terms of the coronary height of about 37.5% (Figure 2F). The PCL membrane did not preclude the formation of the dental crown and root.

### 3.3. Tooth Eruption

**Figure 3:**
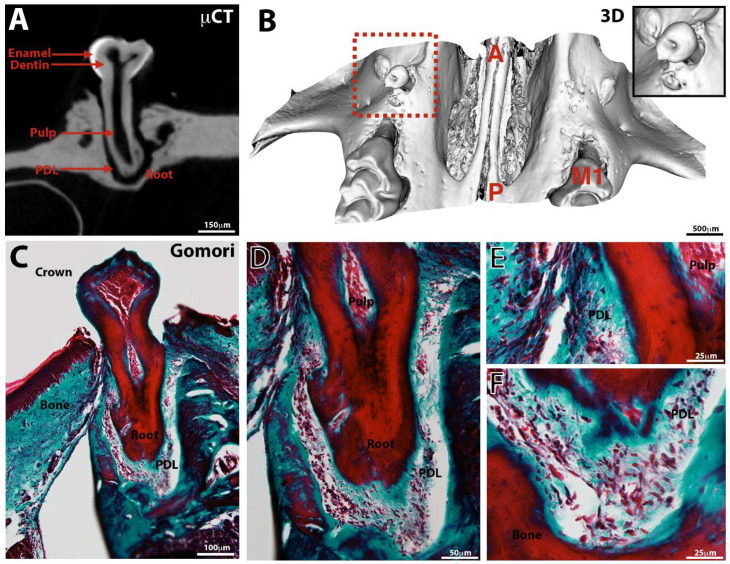
Observation of a non-ankylosed erupted bioengineering tooth. Microtomography (μCT, **A**), 3D reconstruction (**B**) and histology (**C**–**F**, Gomori staining) of a bioengineered tooth implanted with a PCL membrane for 8W in the maxillary bone. The different tissues and structures (root, crown, cusps, apical foramen, enamel, dentin, pulp, periodontal ligament) are present (**A**,**B**), confirmed by histology (**D**–**F**). Vascularized pulp (**D**,**E**,**F**) and PDL are visible (**D**–**F**). A = anterior, M1 = first upper molar, P = posterior and PDL = periodontal ligament.

**Figure 4:**
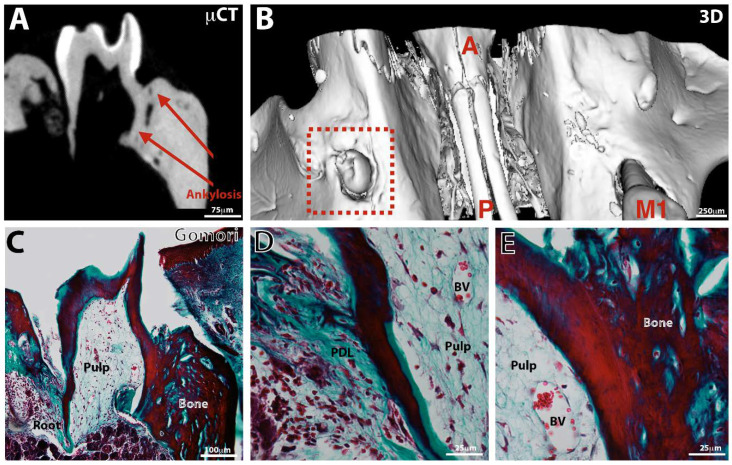
Observation of an ankylosed erupted bioengineered tooth. Microtomography (μCT, **A**), 3D reconstruction (**B**), and histology (**C**–**E**, Gomori staining) of a bioengineered tooth implanted with a PCL membrane for 10W in the maxillary bone. After 8 weeks of implantation, the different tissues and structures (root, crown, cusps, apical foramen, enamel, dentin, pulp, periodontal ligament) are (**A**) confirmed by histology (**C**–**E**). Vascularized pulp (**C**–**E**) and PDL are visible (**D**). Ankylosis is visible. A = anterior, M1 = first upper molar, P = posterior and PDL = periodontal ligament.

At control sites, only one tooth erupted in the oral cavity. Germs implanted with a PCL membrane erupted in 19.2% of cases, as early as the 4th postoperative week (*p* = 0.08) between test and control (Figure 2). Amongst erupted teeth, no pathological mobility (ex vivo examination) and reduced areas of ankylosis (fusion between the bone and the root surface) were observed (Figure 2G,H). A periradicular space can be viewed on the sections obtained by microCT imaging (Figure 3A). Sections from three-dimensional imaging allowed confirmation of the presence of a full, continuous periodontal ligament space (Figure 3A,E,F) or, in some cases, the presence of areas of ankylosis of small extent for the erupted teeth (Figure 4A,C,E). The roots continued their intraosseous formation until apexogenesis (Figure 3C). Early partial ankylosis did not allow a complete eruption of the tooth. In fact, the crown was visible in the mouth, but the occlusal surface was not at the level of those of the adjacent teeth (Figure 4A–D), unlike the implanted teeth, which were not ankylosed (Figure 3A,B).

## 4. Discussion

### 4.1. Tooth Germs Culture and Implantation in Jawbones

When tooth germs are cultivated on a semi-solid medium, structures similar to a dental follicle (DF) develop around the newly formed tooth. Preservation of the DF during and after the stages of culture on a semi-solid medium is an essential element for eruption. Indeed, in previous studies [21], the absence of tooth was observed when DF was resected [21]. This structure is the main regulatory center of molecular interactions involved in the eruption process [21]. Derived from cephalic neural crest cells, the DF surrounds the enamel organ from the cup stage. This connective tissue comprises multipotent mesenchymal stem cells with the potential for differentiation in osteoblasts, adipocytes, chondrocytes, and neural cells [22,23,24]. Their high proliferation rate and immunomodulatory properties make them particularly relevant in tissue engineering [25]. DF cells form the periodontal ligament, cementum, and alveolar bone [24]. The interactions between bone and DF appear to be permitted thanks to the porosity of the nanofibrous structure.

### 4.2. Long-Term Success Assessment

There is a risk of post-eruptive ankylosis. This eventuality would not constitute a failure since the clinical situation would be similar to those obtained with titanium dental implants. In fact, titanium implants are osseointegrated, without peri-implant ligament, which is not a problem when bone growth is complete [26]. The degradation of PCL is slower than that of PGA or PLA [27], this being an advantage as it allows time for the tooth to erupt before the formation of areas of ankylosis.

The membrane did not prevent the development of the tooth, but the size of the bioengineered tooth was smaller than the normal tooth (reduction of coronary height about 37.5%). It can be kept as it is, be crowned to modify its morphology, or even be a support for a prosthetic bridge. Indeed, the erupted teeth appeared to have sufficient bone anchorage to withstand chewing forces. These biomechanical properties have yet to be evaluated.

These preliminary results need to be confirmed over the long term, and the technique improved to better control the spatial orientation of the germ and the success rate for tooth formation and eruption. The main difficulty is related to the small size of the animal.

### 4.3. Comparison with Other Experimental Strategies for Bioengineered Tooth Eruption

In 2011, Oshima et al. [8] developed an approach in which the tooth germ is transiently implanted into a ring-shaped size-control device and then into the subrenal capsule. The main advantages are linked to the formation of bone and periodontal tissues, which form a true bone-tooth unit. This unit was then transplanted into a jaw, directly aligned and occluded with the opposing first molar. This technique is long since the germ was developing in the device for 50 to 60 days, and they observed full bone integration 30 days after implantation in the jawbone. With this technique, several invasive procedures under general anesthesia are necessary, and we can imagine that they are difficult to be accepted in human settings, while in our approach, a single intervention is sufficient for implantation.

### 4.4. Development Prospects for the Nanofibrous Membrane

Future studies could be carried out by functionalizing the PCL membrane with molecules that promote eruption processes. For example, CSF-1 (Colony Stimulating Factor 1) is involved in tooth eruption processes through regulation of the osteoclastogenesis [28,29]. Cielinski et al. showed that the injection of CSF-1, which has a direct effect on the recruitment of osteoclastic cells, accelerates the eruption of molars [30].

Another perspective would be to rely on the RANK-RANKL-OPG signaling pathway. Activation of RANK by binding of RANKL induces the differentiation of monocytes into osteoclasts. RANKL is mainly expressed by osteocytes and osteoblasts [31]. Its expression is modulated during the eruption process: on the 5^th^ day of life in mice, when the activity of osteoclasts is intense in the occlusal region of the germ, there is an important expression of RANKL compared to OPG, and the opposite trend in the basal region [32]. Spatio-temporal control of the molecules delivered artificially around the tooth germ is essential to modulate the osteoclastic activity, and therefore the formation of the eruption path. It is also possible to consider injecting these molecules near the site of interest in order to precisely control the time window of action and the quantity delivered.

## 5. Conclusions

The placement of a PCL membrane to isolate bioengineered tooth germ from alveolar bone allowed tooth morphogenesis and promoted the eruption of the molar in the oral cavity. The porosity of the membrane allows interactions between the tissues involved in the eruption process while limiting early ankylosis. However, failures, mainly related to late ankylosis of the tooth, are still numerous and the functionalization of the membrane with molecules of interest capable of stimulating the eruption is one therapeutic perspective.

## References

[1] Buser D., Sennerby L., De Bruyn H. (2017). Modern implant dentistry based on osseointegration: 50 years of progress, current trends and open questions. Periodontol 2000.

[2] Tenenbaum H., Bogen O., Séverac F., Elkaim R., Davideau J.L., Huck O. (2017). Long-term prospective cohort study on dental implants: Clinical and microbiological parameters. Clin. Oral Implant. Res..

[3] Gómez-de Diego R., Mang-de la Rosa M.R., Romero-Pérez M.J., Cutando-Soriano A., López-Valverde-Centeno A. (2014). Indications and contraindications of dental implants in medically compromised patients: Update. Med. Oral Patol. Oral Cir. Bucal..

[4] Bohner L., Hanisch M., Kleinheinz J., Jung S. (2019). Dental implants in growing patients: A systematic review. Br. J. Oral Maxillofac. Surg..

[5] Schnabl D., Grunert I., Schmuth M., Kapferer-Seebacher I. (2018). Prosthetic rehabilitation of patients with hypohidrotic ectodermal dysplasia: A systematic review. J. Oral Rehabil..

[6] Oshima M., Tsuji T. (2015). Whole Tooth Regeneration as a Future Dental Treatment. Adv. Exp. Med. Biol..

[7] Ma Y., Xie L., Yang B., Tian W. (2019). Three-dimensional printing biotechnology for the regeneration of the tooth and tooth-supporting tissues. Biotechnol. Bioeng..

[8] Oshima M., Mizuno M., Imamura A., Ogawa M., Yasukawa M., Yamazaki H., Morita R., Ikeda E., Nakao K., Takano-Yamamoto T. (2011). Functional tooth regeneration using a bioengineered tooth unit as a mature organ replacement regenerative therapy. PLoS ONE.

[9] Batool F., Strub M., Petit C., Bugueno I.M., Bornert F., Clauss F., Huck O., Kuchler-Bopp S., Benkirane-Jessel N. (2018). Periodontal Tissues, Maxillary Jaw Bone, and Tooth Regeneration Approaches: From Animal Models Analyses to Clinical Applications. Nanomaterials.

[10] Kuchler-Bopp S., Bagnard D., Van-der-Heyden M., Idoux-Gillet Y., Strub M., Gegout H., Lesot H., Benkirane-Jessel N., Keller L. (2018). Semaphorin 3A receptor inhibitor as a novel therapeutic to promote innervation of bioengineered teeth. J. Tissue Eng. Reg. Med..

[11] Kuchler-Bopp S., Larrea A., Petry L., Idoux-Gillet Y., Sebastian V., Ferrandon A., Schwinté P., Arruebo M., Benkirane-Jessel N. (2017). Promoting bioengineered tooth innervation using nanostructured and hybrid scaffolds. Acta Biomater..

[12] Marks S.C., Schroeder H.E. (1996). Tooth eruption: Theories and facts. Anat. Rec..

[13] Lungová V., Radlanski R.J., Tucker A.S., Renz H., Míšek I., Matalová E. (2011). Tooth-bone morphogenesis during postnatal stages of mouse first molar development. J. Anat..

[14] Dwivedi R., Kumar S., Pandey R., Mahajan A., Nandana D., Katti D.S., Mehrota D. (2020). Polycaprolactone as biomaterial for bone scaffold: Review of literature. J. Oral Biol. Craniofac. Res..

[15] Batool F., Morand D.N., Thoma L., Bugueno I.M., Aragon J., Irusta S., Keller L., Benkirane-Jessel N., Tenenbaum H., Huck O. (2018). Synthesis of a Novel Electrospun Polycaprolactone Scaffold Functionalized with Ibuprofen for Periodontal Regeneration: An In Vitro andIn Vivo Study. Materials.

[16] Morand D.N., Huck O., Keller L., Jessel N., Tenenbaum H., Davideau J.L. (2015). Active Nanofibrous Membrane Effects on Gingival Cell Inflammatory Response. Materials.

[17] Xue J., Wu T., Dai Y., Xia Y. (2019). Electrospinning and Electrospun Nanofibers: Methods, Materials, and Applications. Chem. Rev..

[18] Nazarnezhad S., Baino F., Kim H.W., Webster T.J., Kargozar S. (2020). Electrospun Nanofibers for Improved Angiogenesis: Promises for Tissue Engineering Applications. Nanomaterials.

[19] Homaeigohar S., Monavari M., Koenen B., Boccaccini A.R. (2021). Biomimetic biohybrid nanofibers containing bovine serum albumin as a bioactive moiety for wound dressing. Mater. Sci. Eng. C Mater. Biol. Appl..

[20] Stutz C., Strub M., Clauss F., Huck O., Schulz G., Gegout H., Benkirane-Jessel N., Bornert F., Kuchler-Bopp S. (2020). A new polycaprolactone-based biomembrane functionalized with BMP-2 and stem cells improves maxillary bone regeneration. Nanomaterials.

[21] Cahill D.R., Marks S.C. (1980). Tooth eruption: Evidence for the central role of the dental follicle. J. Oral Pathol..

[22] Pan K., Sun Q., Zhang J., Ge S., Li S., Zhao Y., Yang P. (2010). Multilineage differentiation of dental follicle cells and the roles of Runx2 over-expression in enhancing osteoblast/cementoblast-related gene expression in dental follicle cells. Cell Prolif..

[23] Wise G.E. (2009). Cellular and molecular basis of tooth eruption. Orthod. Craniofac. Res..

[24] Zhou T., Pan J., Wu P., Huang R., Du W., Zhou Y., Wan M., Fan Y., Xu X., Zhou X. (2019). Dental Follicle Cells: Roles in Development and Beyond. Stem Cells Int..

[25] Yagi H., Soto-Gutierrez A., Parekkadan B., Kitagawa Y., Tompkins R.G., Kobayashi N., Yarmush M.L. (2010). Mesenchymal stem cells: Mechanisms of immunomodulation and homing. Cell Transplant..

[26] Guillaume B. (2016). Dental implants: A review. Morphologie.

[27] Engelberg I., Kohn J. (1991). Physico-mechanical properties of degradable polymers used in medical applications: A comparative study. Biomaterials.

[28] Grier R.L., Zhao L., Adams C.E., Wise G.E. (1998). Secretion of CSF-1 and its inhibition in rat dental follicle cells: Implications for tooth eruption. Eur. J. Oral Sci..

[29] Rothstein M., Bhattacharya D., Simoes-Costa M. (2018). The molecular basis of neural crest axial identity. Dev. Biol..

[30] Cielinski M.J., Jolie M., Wise G.E., Marks S.C. (1995). The contrasting effects of colonystimulating factor-1 and epidermal growth factor on tooth eruption in the rat. Connect. Tissue Res..

[31] Ono T., Hayashi M., Sasaki F., Nakashima T. (2020). RANKL biology: Bone metabolism, the immune system, and beyond. Inflamm. Regen..

[32] Uribe P., Plakwicz P., Larsson L., Czochrowska E., Westerlund A., Ransjö M. (2018). Study on site-specific expression of bone formation and resorption factors in human dental follicles. Eur. J. Oral Sci..

